# Validation and psychometric testing of the Arabic version of the mental health literacy scale among the Saudi Arabian general population

**DOI:** 10.1186/s13033-023-00615-5

**Published:** 2023-12-05

**Authors:** Nasser F. BinDhim, Nora A. Althumiri, Yasser Ad-Dab’bagh, Mohammed M. J. Alqahtani, Ahmad Kassab Alshayea, Sulaiman M. Al-Luhaidan, Anton Svendrovski, Rashed Abdullah Al-Duraihem, Abdulhameed Abdullah Alhabeeb

**Affiliations:** 1 Informed Decision-Making IDM, Riyadh, 13323 Saudi Arabia; 2 Alfaisal University, Riyadh, Saudi Arabia; 3 Mental Health Department, Neuroscience Center, King Fahad Specialist Hospital-Dammam, Dammam, Eastern Region Saudi Arabia; 4 Department of Psychology, King Khalid University, Abha, Saudi Arabia; 5 Department of Psychology, King Saud University, P. O. Box 2454, 11451 Riyadh, Saudi Arabia; 6 Studies and Information, National Committee for Narcotics Control, P.O. Box 50045, 11523 Riyadh, Saudi Arabia; 7 Department of Community Health and Epidemiology, Faculty of Medicine, Dalhousie University, Halifax, NS Canada; 8 National Centre for Mental Health Promotion, P.O. Box 95459, 11525 Riyadh, Saudi Arabia

**Keywords:** Mental health literacy, Saudi Arabia, Arabic language, Psychometric validation, Cross-cultural adaptation

## Abstract

**Objective:**

This study aimed to validate the Arabic Version of the Mental Health Literacy Scale (Arabic-MHLS) among the Saudi Arabian general population, assessing its internal consistency, test-retest reliability, and structural validity.

**Methods:**

A total of 700 Arabic-speaking Saudi adults were randomly selected to complete the electronic questionnaire in May 2023, which generated 544 participants. Data were coded and stored in the ZdataCloud research data collection system database. Test-retest reliability was assessed using a subsample of 48 participants who completed the questionnaire twice, with a one-week interval. Structural validity was examined using confirmatory factor analysis (CFA) and Exploratory Factor Analysis (EFA).

**Results:**

The Arabic-MHLS demonstrated good internal consistency (Cronbach’s alpha = 0.87) and test-retest reliability (intraclass correlation coefficient = 0.89). EFA revealed a four-factor model closely resembling the model identified in the Slovenian validation of MHLS, with factor loadings ranging from 0.40 to 0.85. The four factors included knowledge of mental health disorders, knowledge of help-seeking, knowledge of self-help strategies, and knowledge of professional help also showed good internal consistency.

**Conclusion:**

The Arabic-MHLS is a valid and reliable tool for assessing mental health literacy in the Saudi Arabian general population. However, further research is needed to refine the measurement tool and understand the complex relationships between mental health literacy and other mental health-related concepts. This will contribute to the development of targeted interventions and policies aimed at improving mental health literacy and promoting mental well-being in the Saudi Arabian population and beyond.

## Introduction

Despite the absence of a universally agreed-upon operational definition for mental health literacy, inadequate mental health literacy has been recognized as a significant impediment to seeking assistance (Gulliver et al. 2010). Enhancements in this area have been linked to decreased personal stigma and increasingly favorable beliefs and intentions regarding professional help-seeking (Jung et al. 2017; Smith and Shochet 2011). Please check and confirm if the authors and their respective affiliations have been correctly identified. Amend if necessary. Specifically, positive attitudes towards the treatment of mental health disorders correlate with more receptive attitudes towards help-seeking and a heightened inclination to utilize mental health services (Altweck et al. 2015; Givens et al. 2007). As a result, numerous interventions aimed at diminishing the treatment gap and improving public mental health have targeted the enhancement of mental health literacy (Kelly et al. 2007; Smith and Shochet 2011).

A plethora of operational definitions for mental health literacy have given rise to a wide range of measurement approaches. Nonetheless, there is a shortage of comprehensive, robust, and psychometrically reliable assessment tools (Krohne et al. 2022; Wei et al. 2016). Many instruments exclusively assess literacy related to specific mental health disorders, such as depression, anxiety, or schizophrenia, while others solely evaluate particular domains of mental health literacy, including knowledge or positive mental health (Amarasuriya et al. 2015; Caldwell and Jorm 2000). Thus, appraising mental health literacy necessitates a tailored approach and interpretation of qualitative responses.

To evaluate mental health literacy in a cost-effective manner and on a larger scale, O’Connor and Casey developed the Mental Health Literacy Scale (MHLS), a robust and quantitative measure encompassing the six facets of mental health literacy identified by Jorm et al. The MHLS is applicable to various mental health disorders and exhibits satisfactory psychometric properties, suggesting its potential use in intervention and evaluation processes (Jorm et al. 1997; O’Connor and Casey 2015). In recent years, further validation studies have emerged, advocating for the MHLS’s application in other linguistic and cultural contexts (El Khalil 2023). Two investigations analyzed the construct validity of the Farsi/Persian-translated scale, leading to modified versions (Heizomi et al. 2020; Nejatian et al. 2021). Previous studies examined the content and construct validity, reliability, internal consistency, and invariance of the Turkish and Slovenian version, while the content validity was also explored within the South African, French, (Korhonen et al. 2019) Slovenia, Chinese and Zambian contexts (Chen et al. 2021; Kesgin et al. 2020; Korhonen et al. 2019; Montagni and González Caballero 2022; Wang et al. 2022; YAVAŞ et al. 2022).

Recently, Saudi Arabia established several periodic mental health surveillance systems and increased the national focus and investment in evidence-based mental health (Althumiri et al. 2022; BinDhim et al. 2021). Saudi Arabia planning to measure the population’s mental health literacy to gain a better understanding of the public’s knowledge, attitudes, and beliefs about mental health issues. Accurate measurement of MHL can help identify gaps in knowledge and misconceptions, which can inform targeted interventions and educational campaigns to improve mental health awareness and reduce stigma (Altweck et al. 2015). This is particularly important since cultural differences may influence beliefs and attitudes about mental illness, as well as help-seeking behaviors (Altweck et al. 2015; Liu et al. 2018). By evaluating MHL across diverse populations, governments and healthcare providers can design more effective and culturally appropriate strategies for promoting mental health and ensuring that individuals receive the appropriate care and support (Altweck et al. 2015; Wei et al. 2015). However, there is a lack of validated mental health literacy measurement tool adapted to the Saudi general population.

Thus, the current study seeks to validate the Arabic (Saudi Arabia adaptation) translation of the MHLS by rigorously scrutinizing its psychometric properties within Saudi’s general population. This research aims to evaluate the structural validity, the scale’s internal consistency and test re-test reliability, and known groups assessment.

## Methods

### Design

Translation of the MHLS from its original version in English to the Arabic language (Saudi Arabia adaptation, followed by, validation study based on two cross-sectional self-reported data collections (samples) of the translated Scale.

### Measures

#### Demographic variables

Participants completed a brief demographic questionnaire that included age, sex, educational level, and a group of questions about history of mental illness (previously diagnosed with mental health condition) or familiarity with mental health like living with a diagnosed relative with mental health condition, or specialization or working in health related fields to examine the differences between groups expected to differ in their MHL (known groups assessment).

#### MHL scale

Afterward the participants were instructed to complete the MHLS, which encompasses 35 items. Respondents evaluated each item utilizing a four-point scale that spans from 1 (Very unlikely = I am certain that it is NOT likely) to 4 (Very Likely = I am certain that it IS very likely) (for instance, “If someone became extremely nervous or anxious in one or more situations with other people (e.g., a party) or performance situations (e.g., presenting at a meeting) in which they were afraid of being evaluated by others and that they would act in a way that was humiliating or feel embarrassed, then to what extent do you think it is likely they have Social Phobia”) or a five-point scale that ranges from 1 (Strongly Disagree) to 5 (Strongly agree) (for example, “Seeing a mental health professional means you are not strong enough to manage your own difficulties”). The MHLS score varies from 35 to 160, where a higher score signifies a sufficient MHL.

#### Translation of the MHLS and item adaptation

In alignment with the guidelines proposed by VD Sousa et al. for the translation and adaptation of instruments for cross-cultural healthcare research (Sousa and Rojjanasrirat 2011), we initiated the process with forward and backward translation, followed by piloting the initial Arabic draft with 10 participants. Each participant evaluated the scale’s instructions and items using a binary scale (clear or unclear). If any component of the instrument was considered unclear, participants were encouraged to suggest revisions for improved clarity. Components deemed unclear by at least 20% of the sample necessitated re-evaluation. The findings indicated that 27 items achieved an agreement level of 80% or higher. The translation team analyzed the remaining 8 items, maintaining 6 as they believed the comments reflected participants’ inadequate knowledge rather than issues with the phrasing. The team made minor modifications to the other 2 items. Lastly, a panel of mental health and research experts assessed and approved the final draft, after rephrasing item 1 to 8, and retesting it on another pilot.

### Participants and data Collection

#### Sample 1: Test re-test reliability

Based on published literature, the recommended sample size for test-retest reliability is 20 to 40 participants (McMillan and Hanson 2014; Walter et al. 1998). In May 2023, 60 randomly selected Arabic-speaking adults from the general Saudi population was invited to complete the questionnaire on an electronic form. Eligibility was determined automatically via the data collection system. The eligibility criteria for participation in the study included being aged 18 years or older and having Arabic as the primary language. Individuals were selected from a national participant database, which contains a representative panel of the Saudi population. Those meeting these criteria were notified through SMS text messages, inviting them to complete the survey via unique survey links. The data collection system employed stratification based on gender to achieve an approximately equal distribution between genders. To enhance response rates, three reminders were sent to each potential participant within a one-week period. If the participant did not respond, another participant with similar demographics was invited until the required sample size had been reached. The same participants completed the questionnaire again after 1 week. Participants were asked to complete all answers before submitting the questionnaire. We used the ZDataCloud research data collection system which also integrates eligibility and sampling modules, to control the sample’s eligibility, distribution and prevent human-related sampling bias, it also include data quality and integrity validation and all questions had to be completed for the response to be successfully submitted to the database. All data were coded and stored in the ZdataCloud database (“ZDataCloud -Research Data Collection, Governance & Quality System,”).

#### Sample 2: Structural validity

The suggested sample size for structural validity includes a minimum of 2 and a maximum of 20 participants per item. For this study, the recommended minimum sample size was 525 participants based on 15 participants at least per each of the 35 items on the MHLS (MacCallum et al. 1999; Schmitt 2011).

In May 2023, a total of 700 Arabic-speaking Saudi adults were randomly chosen, accounting for the possibility of non-responses, to complete the electronic questionnaire. Participants were required to fill in all the answers before submitting the form. The eligibility criteria and recruitment process employed in this phase were akin to those used during the test-retest stage.

### Data analysis

Descriptive statistics were used to describe the sample and the MHL score. Internal reliability of the tool was first checked assuming unidimensionality. Cronbach’s α and McDonald’s test were used to assess internal consistency. The test-retest reliability was assessed with the intraclass correlation coefficient (ICC). Confirmatory factor analysis (CFA) was used to test the hypothesized unidimenstionality. To assess the suitability for conducting the factor analysis, analyses of the correlation between the scale items were conducted using the Kaiser-Meyer-Olkin sample adequacy measure (nonsignificant results mean the data are suitable for factor analysis) and Bartlett test (significant results mean the data are suitable for factor analysis) (Hair et al. 2019; Schmitt 2011). To examine the factorial structure of the scales, an exploratory factor analysis using principal factor extraction was performed. The oblimin rotation, principal axis extraction, parallel analysis was used to obtain clear factorial structures and enable comparison with the other validation studies results, and factors with eigenvalues greater than 1.0 were retained (Hair et al. 2019). Finally, independent sample t-tests were conducted to examine the differences between groups expected to differ in their MHL score (O’Connor and Casey 2015).

## Results

### Sample 1

Of the 48 participants in study stage 1 (for test-retest reliability), 45.8% (22/48) were male and the mean age was 32.6 years (range 21–60). In the analysis of test-retest reliability, the ICC was α = 0.866.

### Sample 2

Sample 2 dataset includes 544 subjects with age ranging from 18 to 72 years, mean age = 32.9, SD = 10.4. There are slightly more females in the sample (n = 308, 56.6%) compared to males (n = 236, 43.4%). In terms of education level 386 (71%) have a bachelor’s degree or above. Furthermore, 189 (34.7%) specialization or working in health related fields, 62 (11,4%) previously diagnosed with mental health condition, and 105 (19.5%) living with a diagnosed relative with mental health condition.

All participants answered all 35 items in the questionnaire, therefore there is no missing data.

The mean score was 115.5 (Standard deviation (SD): 15.2; Range: 66–157; median: 117) out of 160 total possible score (Skewness: -0.29, Kurtosis: -0.05).

Internal reliability of the tool was first checked assuming unidimensionality. Cronbach’s α (0.850) and McDonald’s ω (0.863) values are above 0.80, suggesting very good reliability. This indicates the MHLS tool is possibly unidimensional.

Confirmatory factor analysis (CFA) was further used to test the hypothesized unidimenstionality (Table 1). One-factor CFA model shows the standardized loading estimates to be statistically significant for all except for three items (#12, 15, 23). Standardized loading values range from − 0.17 (item #20) to 0.65 (item #5 and 6), but the average is fairly low (0.37). Only 10 out of 35 items have standardized loading exceeding the suggested threshold of 0.5. These results suggest that it is unlikely that all items in the scale measure the same latent construct.

**Table 1:** Confirmatory factor analysis (CFA)

Factor	Indicator	Estimate	SE	Z	P	Stand.Estimate
Factor 1	Item_1	0.4296	0.0322	13.321	< 0.001	0.5541
	Item_2	0.4833	0.0322	15.022	< 0.001	0.6119
	Item_3	0.4445	0.0339	13.111	< 0.001	0.5476
	Item_4	0.4376	0.0278	15.738	< 0.001	0.6356
	Item_5	0.5014	0.0311	16.139	< 0.001	0.6484
	Item_6	0.5034	0.0309	16.277	< 0.001	0.6509
	Item_7	0.4715	0.0293	16.082	< 0.001	0.6497
	Item_8	0.3701	0.0272	13.594	< 0.001	0.5671
	Item_9	0.3147	0.0335	9.401	< 0.001	0.4077
	Item_10	0.2088	0.0358	5.834	< 0.001	0.2602
	Item_11	0.2825	0.0313	9.016	< 0.001	0.3948
	Item_12	-0.0375	0.0424	-0.886	< 0.001	-0.0404
	Item_13	0.4135	0.0322	12.853	0.376	0.5390
	Item_14	0.3536	0.0377	9.388	< 0.001	0.4078
	Item_15	-0.0194	0.0439	-0.441	0.659	-0.0201
	Item_16	0.6478	0.0506	12.808	< 0.001	0.5401
	Item_17	0.5455	0.0531	10.280	< 0.001	0.4473
	Item_18	0.4542	0.0560	8.113	< 0.001	0.3583
	Item_19	0.5227	0.0523	9.987	< 0.001	0.4353
	Item_20	-0.2039	0.0545	-3.743	< 0.001	-0.1687
	Item_21	0.3625	0.0554	6.548	< 0.001	0.2921
	Item_22	0.5194	0.0495	10.502	< 0.001	0.4509
	Item_23	0.0765	0.0551	1.390	0.165	0.0637
	Item_24	0.4171	0.0579	7.204	< 0.001	0.3218
	Item_25	0.2873	0.0613	4.685	< 0.001	0.2111
	Item_26	0.4164	0.0516	8.062	< 0.001	0.3566
	Item_27	0.4111	0.0574	7.167	< 0.001	0.3186
	Item_28	0.4454	0.0527	8.449	< 0.001	0.3712
	Item_29	0.2614	0.0556	4.704	< 0.001	0.2168
	Item_30	0.4158	0.0570	7.293	< 0.001	0.3322
	Item_31	0.3505	0.0565	6.198	< 0.001	0.2854
	Item_32	0.3319	0.0566	5.860	< 0.001	0.2706
	Item_33	0.1768	0.0522	3.385	< 0.001	0.1564
	Item_34	0.4882	0.0616	7.927	< 0.001	0.3558
	Item_35	0.5404	0.0598	9.042	< 0.001	0.4018

CFA model was further assessed using multiple fit measures. A chi-square test shows a difference between estimated and actual variance-covariance matrix, χ²(560) = 4249, p < 0.001. This suggests the model does not have a good fit. CFI value is fairly low (0.416), well below the threshold of 0.95. RMSEA value is above the required 0.07 value (model RMSEA = 0.110). This further suggests poor model fit. Combined with loa loading estimates, we can say that MHLS does not have a unidimensional factorial structure.

Therefore, Exploratory Factor Analysis (EFA) was be performed to better understand the dimensional structure of the instrument. All 35 items were included into EFA model. Sample size is sufficient for EFA based on Kaiser-Meyer-Olkin test (value 0.865). Barlett’s test of sphericity (χ²(595) = 6745, p < 0.001) is statistically significant, which further confirms that items correlate with each other to the sufficient degree for EFA to be performed.

The initial EFA model (oblimin rotation, principal axis extraction, parallel analysis) has 6 factors. However, the last two factors have very little loading values (mean 0.47 and 0.48), low common variance explained (3.31% and 2.68%) and small eigenvalues (0.55 and 0.35). These factors also have only two items each. Only the first four factors have eigenvalue > 1.

Therefore, the last two factors were excluded and EFA model with 4 factors was constructed (Table 2). It explains cumulatively 37.8% of variability, each factor has eigenvalues > 1. Model overall has good fit with RMSEA = 0.0567 < 0.07. Most items have loading to one (and only one) factor, thus no cross-loading. Only 4 items do not load to any factors (item # 12, 15, 20, 22). Each factor has between 4 and 13 items.

Factor 1 includes items 1–11, 13, 14 which can be labeled as MH recognition. Factor 2 contains items 29–35 that describe attitudes towards people with MH. Factor 3 has items 21, 23–28 which can be interpreted as general attitudes towards MH. Factor 4 has items 16–19 all relating to information seeking about mental illness. High reliability (Cronbach’s α) was obtained for items within each factor: Factor 1 α = 0.857, Factor 2 α = 0.867, Factor 3 α = 0.764, Factor 4 α = 0.809. In addition, high test re-test reliability ICC was obtained for each factor: Factor 1 α = 0.926, Factor 2 α = 0.939, Factor 3 α = 0.819, and Factor 4 α = 0.829. The four-factor model is very similar to model determined in Slovenian validation of MHLS (Krohne et al. 2022).

In terms of known groups assessment, healthcare practitioners (Mean: 121.8, SD:14.1) scored significantly higher than the general population (Mean: 112.2, SD:14.7); t(542)=-7.4 p < 0.001. Moreover, those who were previously diagnosed with mental health condition scored significantly higher (Mean: 122.6, SD:13.9) than those who never been diagnosed with mental health condition (Mean: 114.6, SD:15.1); t(542)=-3.9 p < 0.001. In addition, those who are living with diagnosed relative (Mean: 120.7, SD:14.7) scored significantly higher than those who are not (Mean: 114.3, SD:15.1); t(542)=-3.9 p < 0.001. Moreover, those with bachelor’s degree or above scored higher (Mean: 116.8, SD:14.6) than those with less than with bachelor’s degree (Mean: 112.5, SD:16.3); t(542)=-3.0 p = 0.003. Finally, female (Mean: 118.8, SD:14.4) were significantly higher in mental health literacy score than male (Mean: 111.3, SD:15.1); t(542)=-5.8 p < 0.001.

**Table 2:** Exploratory factor analysis (EFA) with 4 factors

Factor						
	Items	1 MH recognition	2 Attitudes towards people with MH	3 General attitudes towards MH	4 Information seeking about mental illness	Uniqueness
	Item_1	0.556				0.665
	Item_2	0.624				0.586
	Item_3	0.582				0.658
	Item_4	0.647				0.552
	Item_5	0.622				0.553
	Item_6	0.518				0.578
	Item_7	0.743				0.465
	Item_8	0.684				0.570
	Item_9	0.405				0.802
	Item_10	0.302				0.894
	Item_11	0.483				0.741
	Item_12					0.886
	Item_13	0.577				0.643
	Item_14	0.455				0.790
	Item_15					0.947
	Item_16				0.613	0.464
	Item_17				0.727	0.422
	Item_18				0.591	0.629
	Item_19				0.800	0.365
	Item_20					0.899
	Item_21			0.473		0.703
	Item_22					0.780
	Item_23			0.385		0.757
	Item_24			0.501		0.633
	Item_25			0.570		0.687
	Item_26			0.703		0.468
	Item_27			0.621		0.600
	Item_28			0.611		0.569
	Item_29		0.634			0.563
	Item_30		0.757			0.393
	Item_31		0.779			0.385
	Item_32		0.787			0.380
	Item_33		0.609			0.640
	Item_34		0.635			0.549
	Item_35		0.614			0.539

## Discussion

This study involved translating, validating, and psychometrically testing the Arabic Version of the Mental Health Literacy Scale among the Saudi Arabian general population. The Arabic Mental Health Literacy Scale (Arabic-MHLS) demonstrated good internal consistency, test-retest reliability, and generated a four-factor model closely resembling the model identified in the Slovenian validation of MHLS (Krohne et al. 2022). Each of the four factors displayed strong internal consistency. We adopted a factor categorization similar to the Slovenian validation, including mental health recognition, attitudes towards people with mental health issues, general attitudes towards mental health, and information-seeking about mental illness. Like the original MHLS, the Arabic-MHLS effectively distinguished between groups with varying levels of mental health literacy, such as education level, working in health-related fields, previous mental health diagnoses, and living with a relative diagnosed with a mental health condition.

However, unlike the original scale, which was negatively-skewed, the Arabic-MHLS score distribution was nearly symmetrical. This discrepancy may be due to the larger sample size in the Arabic-MHLS and the use of random sampling, as opposed to the snowball sampling employed in the original scale. Other translation and validation studies have focused on specific populations, such as university students (Montagni and González Caballero 2022) or healthcare professionals (Korhonen et al. 2019; Wang et al. 2022), which limits the comparability of our results to general population-based studies. These differences in factorial structure and interpretation may also be attributed to the various study populations.

As noted by Krohne et al., the complex nature of mental health literacy and its association with other extensively researched concepts (e.g., stigmatizing attitudes) led Spiker and Hammer (2019) to propose that mental health literacy is not just a construct but also a theory (Krohne et al. 2022; Spiker and Hammer 2019). Viewing mental health literacy as a theory allows researchers to explore the connections between these concepts and identify the roles that specific constructs play in help-seeking behaviors. To understand these relationships, a comprehensive measurement framework is necessary.

In regard to the cross-cultural application of the Arabic version produced by this study, it is recommended that further validation be considered. Subtle nuances in wording have the potential to significantly affect how statements are interpreted across various cultures. This recommendation is based on insights obtained during the translation process, especially from interactions with focus groups. These interactions underscored the considerable impact that even minor linguistic variations can have.

Lastly, this study is limited by the lack of a universally accepted, psychometrically robust measurement tool (Krohne et al. 2022). Additionally, challenges in defining mental health literacy hinder the validity of the analysis and prevent a thorough examination of the findings. As a result, we recommend that future research investigates the factor structure of the measurement using different sample groups.

## Conclusions

In conclusion, this study contributes to the growing body of literature on mental health literacy by examining the psychometric properties of the Arabic-MHLS in a general Saudi population. The findings highlight the importance of considering cultural and linguistic factors when adapting and validating mental health literacy measures. The Arabic-MHLS demonstrated satisfactory test-retest reliability and structural validity, providing a valuable tool for assessing mental health literacy in Arabic-speaking populations. However, the study is not without limitations, including the lack of a universally accepted measurement tool and challenges in defining mental health literacy. Future research should investigate the factor structure of the measurement using different sample groups and explore the connections between mental health literacy and related constructs, such as stigmatizing attitudes and help-seeking behaviors. By enhancing our understanding of mental health literacy, researchers and practitioners can develop targeted interventions to improve mental health outcomes and reduce the burden of mental illness in diverse populations.

## Data Availability

Available from The National Center for Mental Health Promotion upon request.
